# Five Hot Topics in Tropical Medicine: 2025

**DOI:** 10.3390/idr18030060

**Published:** 2026-06-19

**Authors:** Amanda Hempel, Gregory D. Hawley, Jahmar Hewitt, Maxime Billick, Adrienne J. Showler, Kevin C. Kain, Andrea K. Boggild

**Affiliations:** 1 Department of Medicine, Temerty Faculty of Medicine, University of Toronto, Toronto, ON M5S 3H2, Canada; 2 Tropical Disease Unit, Toronto General Hospital, University Health Network, Toronto, ON M5G 2C4, Canada; 3 Institute of Medical Science, University of Toronto, Toronto, ON M5S 3H2, Canada; 4 Department of Medicine, Georgetown University, Washington, DC 20057, USA

**Keywords:** Chikungunya vaccine, Ebola virus disease, loop-mediated isothermal amplification, malaria, Oropouche virus, snakebite envenoming, Sudan ebolavirus

## Abstract

In 2025, tropical medicine was shaped by advances in diagnostic technology, expanding arboviral epidemics, rapid progress in filovirus vaccine development, evolving regulatory responses to newly licensed vaccines, and translational breakthroughs addressing neglected tropical diseases. This review discusses five select developments that significantly influenced clinical practice and global health policy over the past year, highlighting the implications of each for clinicians, researchers, and public health systems.

## 1. Five Hot Topics in Tropical Medicine in 2025

As we progress through 2026, reflecting on the changes in the landscape of tropical medicine in 2025 allows us to capitalize on the momentum of successes and identify challenges requiring renewed efforts. Neglected tropical diseases (NTDs) along with malaria continue to burden over 1 billion people globally and exact an exorbitant toll economically and from a health equity and human rights perspective given their differential impact in the tropics. In this review, we highlight five of the many potential hot topics that made an impact on the field of tropical medicine in 2025. We selected the topics based on crowd-sourced feedback from the local group of expert staff physicians and Tropical Medicine fellows providing clinical service in our ambulatory Tropical Disease Unit at the largest academic hospital in Canada. We particularly focused on Hot Topics that are relevant to our scope of practice in a North American context. As such, emerging issues of particular significance to other geographic regions were not included in our “Top Five”. Table 1 lists our selected Top Five Hot Topics along with notation of the issues that emerged in 2025.

## 2. Hot Topic 1: Loop-Mediated Isothermal Amplification for Malaria Diagnosis

*Scope of impact*: global.

Malaria remains endemic in 83 countries and continues to represent a leading cause of fever amongst travelers in non-endemic countries [1]. Microscopy is the mainstay of diagnosis as it provides species identification and quantification of parasitemia [2,3,4,5]. bHowever, it requires expertise and equipment (e.g., staining, microscopes) and has a limit of detection (LOD) of 50–200 parasites/μL [2,4]. Rapid diagnostic tests (RDTs) are immunochromatographic point-of-care tests that have a turnaround time of 2–15 min but have an LOD of 200 parasites/μL and low sensitivity for non-falciparum species [2,4]. Nucleic acid amplification tests (NAATs) are far more sensitive. Polymerase chain reaction (PCR) is the reference molecular standard, with a LOD ranging from 0.1–5 parasites/μL, but it is expensive, requires both specialized equipment and trained personnel, and has a turnaround time of hours to days, all of which prevent its use outside of reference labs [3,4,5,6].

### 2.1. Emergence of LAMP as a Molecular Diagnostic Tool

Loop-mediated isothermal amplification (LAMP) is a single-temperate single-tube amplification technique [3,4,7]. It produces results in <1h and requires only basic equipment and limited technical training [3,7,8]. Over 40 malaria LAMP assays have been described, and two commercialized assays are currently available [3,7]. LOD ranges from 1–5 parasites/μL, with sensitivity approaching that of PCR and nearly 100% specificity [3,4,7]. LAMP is far more sensitive than RDT or microscopy but is cheaper, simpler and faster than PCR. However, assays do not quantify parasitemia and remain positive up to 4 weeks post-treatment and, as such, cannot discriminate between clinically irrelevant sexual parasitemia (i.e., gametocytemia) and highly clinically relevant asexual parasitemia (i.e., ring stages, trophozoites) [4,9,10]. Although LAMP can be used to differentiate species, its ability to do so depends on the primers in the assay [3,7,8]. After generating significant interest starting in the mid-2010s, research has been focused on the best way to utilize LAMP.

### 2.2. What Is New?

In non-endemic countries, malaria diagnosis generally requires three negative blood smears to rule out infection. This generates a significant burden of labor for laboratories, and expertise can be difficult to maintain [7,11,12]. Multiple studies from 2017–2021 reported a high sensitivity of LAMP for malaria in returning travelers and recommended a novel algorithm with LAMP as the initial test, where a single negative LAMP is sufficient to rule out malaria and a positive LAMP prompts microscopy for confirmation, species identification, and parasitemia quantification [12,13,14,15,16]. This can save hundreds of hours of technologist time per year and reduce costs per patient [12,17]. Since 2021, successful implementation of this algorithm has been reported in multiple settings [11,18,19,20]. Although not yet mentioned in the United States Centers for Disease Control and Prevention (CDC) malaria guidelines, LAMP was added to the British Society of Hematology guidelines in 2022, and the single-LAMP rule-out algorithm was included in the Canadian Committee to Advise on Travel and Tropical Medicine (CATMAT) guidelines in 2023 [4,5]. By 2025, this alternative LAMP algorithm saw increased uptake in multiple countries. At our center, LAMP was implemented in 2025 as part of the screening procedures for malaria.

In contrast, LAMP has seen limited use in endemic countries. LAMP is neither equipment-free nor affordable for point-of-care settings [7,8,21]. It is also not clear when the improved LOD is actually of benefit in endemic settings. Prior studies did not find improved clinical outcomes in PCR-positive, RDT-negative cases, though studies on chronic complications are lacking [21]. Malaria in pregnancy is associated with low parasitemia and severe birth outcomes; however, while a recent clinical trial in Ethiopia did find LAMP more sensitive than comparator assays, it was underpowered to detect a difference in birthweight [21,22]. From a public health perspective, low-parasitemia infections are relevant as they contribute to forward transmission, and prevalence studies would benefit from the increased sensitivity of nucleic-acid testing [7,21,23]. Moreover, modeling suggests that mass test-and-treat programs with an ultrasensitive point-of-care test could accelerate elimination [23]. Currently, the World Health Organization (WHO) does not recommend LAMP as a viable option for point-of-care, particularly given the lack of evidence that detecting low-level parasitemia leads to improved outcomes [6]. However, numerous recent studies have proposed modifications to LAMP that would decrease the cost and simplify use for point of care, including the use of dried blood spots, reducing the amount of reagent, simplifying read-out methods, shortening incubation times and increasing capacity per run [23,24,25,26,27]. Future studies can focus on determining if these adjustments have a significant impact on clinical and public health outcomes.

**Practice Implications for Clinicians**: Unlike thick and thin blood smears and RDT, which require multiple specimens collected over time, a single LAMP assay is sufficiently sensitive to serve as a “rule-out” test for malaria after one blood collection. While LAMP assays are highly advantageous for reasons of rapidity and parsimony, they are limited by their inability to differentiate between clinically relevant asexual blood stages and clinically irrelevant gametocytemia, as well as their non-quantitative readout. For these reasons, especially in non-endemic areas, LAMP should be supplemented by highly sensitive and specific composite algorithms (e.g., thin smear plus RDT) or PCR.

## 3. Hot Topic 2: Oropouche Virus Expansion in the Americas

*Scope of Impact*: predominantly regional, Central and South America.

Oropouche Virus (OROV) is a single-stranded negative-sense RNA virus that is part of the Orthobunaviridae family [28]. Since its discovery in the Oropouche Valley of Trinidad and Tobago in 1955, there have been sporadic outbreaks infecting humans in Central and South America [29]. Outbreaks have typically been limited to the Amazon Basin [30,31]. However, a multinational outbreak in 2024–2025 was unprecedented in both scope and presentation and brought new attention to the virus.

Oropouche virus is predominantly transmitted via a bite from the *Culicoides paraensis* midge and occasionally from mosquitoes (*Culex* and *Aedes*) [31,32]. *C. paraensis* is a poor flier and breeds in shallow stagnant pockets of water and detritus, making decomposing cacao husks and banana and plantain stalks prime reproductive locations [31]. It is believed that significant Amazonian deforestation and widespread banana plantations have contributed to OROV’s spread. OROV has both a sylvatic cycle involving birds and apes as well an urban cycle predominantly affecting humans [31,32].

### 3.1. Clinical Presentation

The OROV incubation period lasts 3–10 days after infection [31,32]. Common symptoms are dengue-like and include an abrupt onset fever, headache, retro-orbital pain and photophobia, arthralgias, myalgias, nausea and vomiting, and a rash that may or may not be pruritic [32,33]. Rare presentations include aseptic meningitis, encephalitis, and hemorrhagic manifestations [32,33]. Most people experience symptoms for 2–7 days, but symptoms may persist for 2–4 weeks if severe. Interestingly, there are some reports of up to 60% symptom recurrence 1–2 weeks later [32,33,34]. Lab findings are poorly characterized but can include elevated liver enzymes and leukopenia [32,33,34]. Treatment is supportive and includes rest, judicious fluids, acetaminophen, and avoidance of non-steroidal anti-inflammatory medications due to risk of hemorrhage [35]. No specific antiviral therapies, monoclonal antibodies, or licensed vaccines are currently available for the treatment or prevention of Oropouche infection., As such, prevention is largely limited to vector control (insecticides), protective clothing, and insect repellents [34,35].

### 3.2. What Is New?

The 2024–2025 OROV outbreak was unique for a multitude of reasons. First, the number of infections far exceeded that of prior outbreaks; over 16,000 confirmed cases in 2024 and over 12,000 confirmed cases in 2025, as of August 2025 [36]. Furthermore, the territory affected is vast, spanning from Cuba, Barbados and Panama, to Peru, Chile and Bolivia, with the overwhelming majority of cases reported in Brazil (>85%) [36]. Importantly, there has been local acquisition and autochthonous spread in non-Amazonian Brazilian states, suggesting transgression of prior boundaries [37].

Perhaps the most notable difference with the 2024–2025 outbreak is the presence of severe, life-threatening presentations not previously recognized. The first two confirmed adult deaths associated with OROV infection were documented in this outbreak [38]. The deaths occurred in two young Brazilian women, ages 21 and 24 years, following brief febrile illnesses with rapid deterioration and bleeding [38]. There were also several cases of vertical transmission (mother to unborn fetus) resulting in stillbirths, miscarriage, and microcephaly, with likely many more unreported [33,35,36,37,38,39]. Prolonged shedding of replication-competent OROV was further noted in body fluids such as whole blood and semen, [40] which has implications for secondary transmission and infection prevention and control practices.

Furthermore, the 2024–2025 outbreak highlighted challenges with OROV recognition and diagnosis. Clinically, OROV is indistinguishable from dengue, chikungunya, and Zika, which are commonly clinically diagnosed; thus, OROV outbreak cases may actually be underestimated. Current options for microbiologic diagnosis include virologic detection, such as reverse transcription quantitative polymerase chain reaction (RT-qPCR), and serologic testing, including the plaque reduction neutralization test (PRNT) and IgM antibody-capture enzyme-linked immunosorbent assay (MAC-ELISA) [41,42]. RT-qPCR can be run on a multitude of samples (serum, blood, CSF, viscera) and detects the virus itself, but it is only effective within the first 5–7 days of symptom onset [41,42]. PRNT is specific and sensitive in that it measures the titer of neutralizing antibodies for the virus, but it is a time-consuming and expensive test and is unlikely to be practical in low-resource settings. MAC-ELISA is faster and easier to replicate than a PRNT; however, it can have cross-reactivity with similar prior infections, limiting its use in endemic areas. Both PRNT and MAC-ELISA can be performed beyond 5–7 days [41,42]. All such tests would be considered reference-level diagnostics and, outside of endemic areas, are only likely to be available through national reference centers.

The 2024–2025 Oropouche outbreak represented a significantly expanded epidemiology for the previously underrecognized virus, necessitating increased awareness amongst both local health care providers and potentially international providers caring for travelers to the regions. The clinical and geographic overlap with other arboviruses in the region provides a particular challenge to diagnosis.

**Practice Implications for Clinicians:** Oropouche should be considered in the differential diagnosis of ill travelers returning from areas of ongoing outbreaks, in particular in those with dengue-like symptoms. Many such patients will end up being screened not just for dengue but also *Aedes*-vectored Zika and chikungunya, and Oropouche represents yet another arbovirus presenting in a sufficiently non-specific manner as to warrant inclusion in an expanding differential diagnosis. Testing for Oropouche may be challenging to access and available only through national reference-level laboratories. Clinicians should therefore consult their regional reference laboratory for the most up-to-date specimen collection and processing information when caring for such patients.

## 4. Hot Topic 3: Ebola Virus Disease and Vaccine Trials

*Scope of Impact*: predominantly regional, Central and East Africa.

Four species of ebolaviruses are known to cause human Ebola Virus Disease (EVD): Ebola or Zaire (EBOV), Sudan (SUDV), Bundibugyo (BDBV) and Tai Forest (TAFV) [43,44]. For decades after Ebola was first recognized in 1976, small outbreaks occurred in remote forested areas of Central Africa (EBOV) and East Africa (SUDV) [43,44]. However, in 2014–2016, major outbreaks of EBOV occurred in urban areas of West Africa, with over 28,000 cases, and a few other outbreaks of EBOV—both large and small—occurred in the years since [43,44]. During these outbreaks, EBOV has been noted not only for its high fatality rate and significant chronic sequelae but also persistence of the virus in immune-privileged sites [43,45,46,47,48].

Two monoclonal antibodies and two vaccines are currently licensed for EBOV [49,50,51]. During the 2018–2020 outbreak in the Democratic Republic of Congo, the WHO and the Institute National De Recherche Biomedicale obtained approval to initiate ring vaccination with the ZEBOV vaccine [52]. ZEBOV (rVSV-ZEBOV-GP) is a recombinant vesicular stomatitis virus modified to express the EBOV surface glycoprotein [52]. Ring vaccination involves vaccination of all close contacts of cases as well as close contacts of the contacts. Over 265,000 participants were vaccinated to form almost 2000 rings, and a significant decrease in EVD incidence was noted 10 days post vaccination [52]. Additionally, the case-fatality rate amongst vaccinees who acquired Ebola disease was 23% compared to the outbreak’s overall case-fatality rate of 75% [52].

### 4.1. Limited Therapeutic Options for Sudan Ebolavirus

In contrast, we have much less knowledge about SUDV because there have been only nine outbreaks, with significantly lower case numbers. Neither vaccines nor monoclonal antibodies against EBOV provide cross-protection for SUDV [49,50,53,54]. There are no licensed vaccines or therapeutics, although a few candidates are in development or pre-clinical studies [55,56,57,58,59,60,61,62].

### 4.2. What Is New?

From January to April 2025, Uganda experienced a SUDV outbreak of 14 cases [54]. Although small, this outbreak was notable because within four days of the first case, Uganda launched the first-ever clinical efficacy trial for a SUDV vaccine [63]. Similar to ZEBOV, the SEBOV vaccine (rVSV-SUDV-GP) is a recombinant live vesicular stomatitis virus modified to express the SUDV surface glycoprotein [61]. A previous phase I trial demonstrated humoral immune responses at day 28, with no serious adverse reactions [58]. Since 2023, the WHO has pre-positioned vaccines in Uganda for ring vaccination as part of the TOKEMEZA SVD trial by Makerere University Lung Institute [54,63,64]. During the 2025 outbreak, 17 rings were formed and randomized at an unprecedented speed [65,66]. However, with only 14 cases and rapid control, the full scope of the ring vaccination trial and use of other pre-positioned therapeutics during that outbreak remain unpublished and require additional data. If data do emerge from this trial, they will certainly inform next steps in the development of the SEBOV vaccination and therapeutics strategy.

Several studies also furthered our knowledge about the sequelae of SUDV. A two-year follow-up of survivors of the 2022–2023 outbreak found approximately half of survivors had ongoing generalized, musculoskeletal or neurologic symptoms, and 20% had ongoing ophthalmologic symptoms [67]. A study of survivors of the 2000 Sudan ebolavirus outbreak reported ongoing symptoms of joint pain (36%), visual impairment (36%), fatigue (18%) and neurologic symptoms (13%) more than 25 years after infection [68]. Compared to EBOV, auditory symptoms are not common in SUDV survivors, whereas neurologic sequelae appear more common (e.g., memory loss, numbness and depression) [67].

Furthermore, we have increasing evidence of SUDV persistence in immune-privileged sites. Non-human primates that survived SUDV infection were found to have persistence of the virus in the eyes and testes after 30 days [69], The study of 2022–2023 outbreak survivors found 48.5% of males had positive RNA PCR in semen, with a median duration of positivity of 131 days [67]. In two patients, SUDV RNA was detected in the semen after several months of negative results, suggesting latency and reactivation of the virus [67]. All four lactating participants had positive PCR from breastmilk, with a median duration of positivity of 149 days [67]. This highlights the importance of follow-up of survivors to reduce the risk of recrudescent infection or reignition of outbreaks. Interestingly, genomic sequencing of the 2025 outbreak strain suggested it was closely related to the 2012 outbreak strain, suggesting either persistent circulation in reservoir animals or cryptic transmission in the community from persistent or asymptomatic infections [70].

On 15 May 2026, the Ministry of Public Health, Hygiene and Social Welfare of the Democratic Republic of the Congo (DRC) declared its seventeenth outbreak of Ebola virus disease, while the Uganda Ministry of Health simultaneously declared an outbreak due to an imported case of Bundibugyo virus disease from the DRC [71]. Two days later, the WHO declared that the Bundibugyo virus disease (BVD) outbreak in DRC and Uganda constituted a public health emergency of international concern (PHEIC) [71]. Given that the DRC outbreak is situated in remote, severely under-resourced, and unstable regions of the northeastern part of the country besieged by violence, insecurity, humanitarian crises, high population mobility, and community mistrust, disease notification was significantly delayed, and control efforts (particularly contact tracing) were severely hampered [72]. There are no currently approved or licensed therapeutics of preventive vaccines for BVD [73]. As of 11 June 2026, there have been 695 confirmed cases (676 in DRC and 19 in Uganda) and 138 deaths (136 in DRC and 2 in Uganda), with 37 individuals fully recovering from BVD (32 in DRC and 5 in Uganda) [74]. The true scale of the outbreak likely far exceeds these reported numbers, which do not account for suspected cases awaiting laboratory confirmation.

**Practice Implications for Clinicians**: Beyond the obvious need for strict adherence to infection prevention and control protocols when encountering patients with suspected viral hemorrhagic fever and the necessary reliance on experimental therapeutics in the care of ebolavirus patients, in general, the evolving knowledge around SUDV survivorship echoes that of EBOV, notably around prolonged shedding of viral RNA in body fluids such as semen and breastmilk. As such, ongoing surveillance, counseling, and secondary prevention interventions are now part of our standard approach to care.

## 5. Hot Topic 4: The IXCHIQ Chikungunya Vaccine

*Scope of Impact*: global.

Chikungunya virus (CHIKV) is an arbovirus transmitted by *Aedes* mosquitoes that commonly presents as an acute febrile illness with arthralgias, classically a severe symmetric polyarthralgia of the small joints. A subset of patients may go on to develop a chronic, debilitating polyarthritis similar to rheumatoid arthritis. Sporadic urban outbreaks occurred in Africa and Asia until 2004, when a new epidemic strain caused major outbreaks spreading across Africa, the Indian subcontinent and Southeast Asia; by 2013, local transmission was established in the previously non-endemic Americas [75,76,77,78,79,80,81,82]. In 2024–2025, there was a resurgence of CHIKV, with 445,271 suspected and confirmed cases between January and September 2025 [83].

### 5.1. Development of the IXCHIQ Vaccine

IXCHIQ is a live attenuated, single-dose vaccination with 96.8% seroprotection at 2 years (95% CI 94.3–98.5) [84]. Initial safety data were collected primarily through a Phase III clinical trial that enrolled 4115 patients [85]. Notably, only 11% of participants receiving the vaccine were aged >65 years, and only 2% were >75 years of age. In the vaccine group, 50.4% experienced systemic adverse events, compared to 27% in the placebo group, with the most commonly reported effects including headache, fatigue, myalgia, arthralgia, fever, nausea, rash and vomiting [85]. More than 95% of adverse effects resolved within 2–5 days. However, a subset of patients developed Chikungunya-like adverse reactions defined as fever ≥38 °C and at least one of arthralgia/arthritis, myalgia, headache, back pain, rash, lymphadenopathy or certain ocular, cardiac or neurologic symptoms. Chikungunya-like adverse reactions occurred in 11.7% of the vaccine group, with severe adverse reactions (SAEs) occurring in 1.6% and symptoms lasting >14 days in 0.7% [85].

In November 2023, the United States Food and Drug Administration (FDA) granted accelerated approval to license IXCHIQ in the USA for individuals ≥18 years old but included a warning that the vaccine may cause severe or prolonged chikungunya-like adverse reactions [86]. In June 2024, IXCHIQ subsequently received authorization both by Health Canada and the European Medicines Agency (EMA) [87,88].

### 5.2. What Is New?

In 2024, the Vaccine Adverse Event Reporting System (VAERS) identified cardiac and neurologic events following vaccination in individuals >65 years of age. In April 2025, the Advisory Committee on Immunization Practices (ACIP) meeting reviewed 28 adverse events and identified six cardiac or neurological SAEs, all occurring within 3–5 days of vaccination in males ≥65 years of age, most with multiple comorbidities [89,90]. The meeting resulted in the removal of the recommendation for IXCHIQ in older individuals. By May 2025, the EMA reported a global total of 17 SAEs, including two deaths, in individuals ≥65 and recommended a temporary pause on the use of IXCHIQ in individuals ≥65 [91]. Also in May 2025, the FDA recommended a pause in individuals ≥60 years of age, while the CATMAT recommended against use in individuals ≥65 years of age [9,92].

In July 2025, the EMA lifted the temporary restriction in ages ≥65, concluding that for all ages vaccination with IXCHIQ should only be considered where there is significant risk of infection and a careful risk/benefit assessment [93]. As of 31 August 2025, there have been 35 reported SAEs related to IXCHIQ vaccination [94]. The median age of SAEs was 73 years (range 28–89), with 27 (77%) occurring in adults ≥65 [94]. Twenty-seven cases were male (77%), while 32 cases (91%) had comorbidities and 29 cases (83%) had multiple chronic medical conditions [94]. Three fatalities were reported, with one case of encephalitis with vaccine-strain CHIKV detected in CSF and plasma [94,95]. In three cases of encephalitis—ages 84 to 85 years—on the island of La Réunion (France) following live-attenuated CHIKV vaccination, autoantibodies neutralizing type I interferons were detected [96].

By October 2025, over 80,000 doses of IXCHIQ had been distributed globally [97].

Going into 2026, IXCHIQ remains licensed in the Europe Union, the UK and Canada, although it is currently suspended in the USA. In these regions, CHIKV vaccination exists primarily for individuals travelling to regions with ongoing CHIKV transmission, and careful risk/benefit analysis must consider risk of exposure against risk of adverse events from the vaccine, particularly considering an alternative vaccine, Vimkunya, is available in the European Union, UK and USA and under review in Canada. Vimkunya, a virus-like particle (VLP) single-dose chikungunya vaccine developed by Bavarian Nordic was approved by the FDA on 14 February 2025 for use in individuals 12 years of age and older. As such, a viable alternative to IXCHIQ in many areas, and likely in Canada soon, exists. However, in regions where CHIKV is highly endemic or experiencing outbreaks, IXCHIQ may have a net benefit even in older adults, and further surveillance data are necessary to evaluate ongoing use of the vaccine.

**Practice Implications for Clinicians**: Vimkunya is a single-dose VLP vaccine that is already licensed and marketed in many regions and may serve as an alternative to IXCHIQ in those at risk of clinically important adverse events. Persons with autoantibodies neutralizing type I interferons should not receive the live-attenuated CHIKV vaccine, IXCHIQ.

## 6. Hot Topic 5: Universal Antivenom for Snakebite Envenoming

*Scope of Impact*: global, predominantly affecting tropical and subtropical regions.

Approximately 5.4 million snakebites occur every year, with between 80,000 and 137,000 deaths and 400,000 major disabilities [98]. However it receives limited attention partly due to a disproportionate impact in low- and middle-income countries, particularly in rural and tropical areas. For this reason, the WHO declared snakebites a neglected tropical disease (NTD) and set a target to halve deaths by 2030 [99].

### 6.1. Limitations of Conventional Antivenoms

The mainstay of treatment is antivenom, which involves hyperimmunizing a horse with venom and collecting the serum immunoglobulin for human administration [100]. While it can be effective, it has numerous drawbacks. Non-human serum both causes hypersensitivity reactions and requires cold-chain storage [100]. But most importantly, antivenoms are highly specific to the snake. Snake venom can contain 5–70 protein toxins, and the exact “cocktail” can differ significantly not only between species but even between members of a single species from different regions [100]. Immunoglobulin against one venom may offer limited cross-protection against another [100,101]. In regions with many snake species, not only do centers have to stock multiple antivenoms but the provider must determine the species that bit the patient [101]. Even then, antivenom may have variable regional efficacy. Furthermore, the narrow use for an individual anti-venom and the primarily low-income patients create no market incentive to produce antivenoms [100,102]. Altogether this creates a situation where antivenoms are hard to come by, difficult to use and complicated by serious side effects.

### 6.2. What Is New?

In May 2025, Glanville and colleagues published the first step towards a broad-spectrum antivenom [100]. The authors theorized that venom proteins must have conserved epitopes at the binding site to maintain their effect, and exposure to a broad range of venom would select for antibodies that protect against these preserved epitopes to provide broad protection [100]. This brought them to Tim Friede, who had been bitten 856 times between 2001–2018 by a variety of snakes [100]. Glanville and colleagues took Tim’s donor serum and isolated the broadly neutralizing antibodies into an “immune library” to be used for future studies [100].

This study focused on creating a universal antivenom against elapid snakes by identifying the minimum number of toxins one must protect against to achieve broad spectrum protection. They selected 19 representative elapid species to develop a venom panel [100]. They sequentially identified either a broadly neutralizing antibody or small molecule for the most common toxins and added them to a cocktail until it protected against most elapid venoms. The final cocktail consisted of three components against the three most common toxins—the broadly neutralizing human antibodies LNX-D09 and SNX-B03 and the small molecule varespladib [100]. Out of the 19 representative elapids, the universal antivenom provided complete protection against 13 snakes and partial protection against six and can be presumed to provide broad coverage against most elapids [100].

This study achieved multiple goals. It created a human immune library for antibodies against venom toxins that can be used in future antivenom development and identified two broadly neutralizing antibodies against two of the most common elapid toxins. It developed a cocktail that provided in vivo protection against 19 elapids from the WHO-identified snakes of greatest concern. But perhaps most importantly, it provided the proof of concept and methodology for the development of universal antivenoms.

Why is this a game changer? Universal antivenom reduces the need for difficult species identification, provides consistent coverage across geographical areas, and permits stockpiling [100]. As a human-derived antivenom, it is both safer for patients and reduces cold-chain requirements [100], which increases ease of use and implementation feasibility and improves the business case for the production of antivenoms. This may be the first step in creating a treatment that is both effective and accessible to the most impacted communities.

**Practice Implications for Clinicians**: Snake-specific anti-venoms are poorly tolerated, inaccessible, and require cold-chain supply. A “universal” serum based on human-derived neutralizing antibodies would enable stockpiling and reduce the waste and inefficiency associated with storing snake-specific anti-venom. This could lead to greater population reach and implementation fidelity in the envenomed population globally and thereby save lives.

## 7. Discussion

We crowd-sourced five Hot Topics in tropical medicine from our local group of expert staff physicians and Tropical Medicine fellows providing clinical service in our ambulatory Tropical Disease Unit in order to provide a timely update on evolving issues of clinical importance to our field in 2025. Such topics represented emerging pathogens such as Oropouche and Sudan ebolavirus, newly implemented detection methods at our center including LAMP for malaria, evolving regulatory developments related to the Health Canada licensed Chikungunya vaccine, and updates around the perennial hot topic of snake envenomation. We did not exhaustively or systematically search the literature to arrive at our list of Hot Topics, and as such, experts practicing tropical medicine in other geographic locations, those at different career stages with differing areas of accountability, and those conducting primarily field-based or basic science research may have subjectively different opinions about which topics were the “hottest” of 2025. As such, a comprehensive review of all emerging issues in the field was not undertaken, and for this reason, a topical review of 2025 issues, such as the notifications of Crimean Congo Hemorrhagic Fever (CCHF) in Spain and Greece [103], was not included herein. This development, in particular, strongly aligns with the scope of this review in terms of vector ecology, climate change, travel medicine, laboratory preparedness, and the geographical expansion of tropical and zoonotic diseases in Europe. In brief, as of 8 October 2025, three cases of CCHF have been reported from Spain and two from Greece, one of which was fatal [103]. While prior human cases had occurred in several regions of Spain, and the Nairovirus (family Bunyaviridae) is known to be present in livestock populations there, the cases in Greece were unexpected. The European Centers for Disease Control and Prevention (ECDC) defines CCHF as an emerging tickborne infectious disease in Europe and states that the case fatality rate can range from 5 to 40% in outbreaks [104]. CCHF is therefore an emerging disease of high consequence and significant public health relevance.

Our review describes five select developments that substantially influenced clinical practice and global health policy over the year 2025 and highlights the implications of each for clinicians, researchers, and public health systems. Given our practice location, our selection of key Hot Topics may not generalize to all clinicians practicing tropical medicine in all locations.

## 8. Conclusions

Tropical medicine remains a dynamic field with many eventful developments in 2025, including but not limited to outbreaks of emerging infectious diseases, evolving ideas on how best to deploy newer malaria diagnostics, steps forwards and backwards in vaccines, and new directions for creating anti-venoms. These successes and challenges should motivate ongoing attention to emerging and neglected tropical diseases as well as consideration of how tests and vaccines may be deployed differently in endemic areas versus in travelers from non-endemic regions.

## Figures and Tables

**Table 1:** Top five hot topics in tropical medicine over 2025.

Hot Topic of 2025	Scale of Impact	Type of Emerging Issue	Clinical Implications
Malaria diagnostics	Global	Implementation of LAMP for initial diagnosis of malaria Affects: laboratory diagnostic algorithms; clinical care	A single LAMP test is sufficient to rule out malaria and does not require repeat testing within 24–48 h, unlike traditional microscopic smears and antigen-based RDT
Oropouche virus	Regional	Outbreak of Oropouche virus in the Americas Affects: epidemiological surveillance; clinical care	Clinicians encountering patients with dengue-like illness in travelers to the tropical Americas should consider Oropouche on the differential diagnosis
Ebola virus disease	Regional	Outbreak of Sudan ebolavirus in Uganda Affects: global public health preparedness; infection prevention and control; clinical care	Survivors of Sudan ebolavirus report ongoing clinical sequelae and have been found to shed detectable viral RNA in semen and breastmilk long after convalescence
Live attenuated Chikungunya vaccine (IXCHIQ)	Global	Severe adverse events following IXCHIQ administration in persons over age 65 years led to regulatory amendments and FDA withdrawal of vaccine approval Affects: public health outbreak response; clinical care	For all ages, IXCHIQ should only be considered where there is significant risk of infection and a careful risk-to-benefit evaluation has been undertaken
Snakebite	Global	Using the donor serum of a frequently envenomed person who produces broadly neutralizing antibodies, scientists are on the pathway to creating a universal antivenom against elapid snakes Affects: pharmacy formularies; emergency preparedness; clinical care	Snake-specific anti-venoms are poorly tolerated, inaccessible, and require cold-chain supply. A “universal” serum based on human-derived neutralizing antibodies could lead to greater population reach and implementation fidelity in the envenomed population globally

Abbreviations: FDA, United States Food and Drug Administration; LAMP, loop-mediated isothermal amplification; RDT, rapid diagnostic test.

## Data Availability

No new data were created or analyzed in this study. Data sharing is not applicable to this article.
